# Triple-M Overlap Syndrome Associated with Immune Checkpoint Inhibitors: A FAERS Pharmacovigilance Analysis

**DOI:** 10.3390/healthcare14111466

**Published:** 2026-05-26

**Authors:** Bader Alshamsan, Terry L. Ng

**Affiliations:** 1 Department of Medicine, College of Medicine, Qassim University, Buraydah 52571, Saudi Arabia; bshmsan@qu.edu.sa; 2 Department of Medicine, The Ottawa Hospital Cancer Centre, Ottawa, ON K1H 8L6, Canada; 3 Division of Medical Oncology, Department of Medicine, University of Ottawa, Ottawa, ON K1H 8L6, Canada; 4 Cancer Research Program, Ottawa Hospital Research Institute, Ottawa, ON K1H 8L6, Canada

**Keywords:** immune checkpoint inhibitors, myasthenia gravis, myositis, myocarditis, Triple-M syndrome, pharmacovigilance, FAERS, immune-related adverse events

## Abstract

**Highlights:**

**What are the main findings?**

- Co-reporting of myasthenia gravis, myositis, and myocarditis was common in FAERS, with 21.9% of reports involving at least two components of the triad.
- Triple-M cases showed early onset (median 21 days) and substantial reported fatality (50% among cases with known outcomes).

**What are the implications of the main findings?**

- Detection of one component may prompt early evaluation of the other components.
- These findings highlight the importance of multidisciplinary management and close clinical monitoring.

**Abstract:**

**Background/Objectives**: Immune checkpoint inhibitors (ICIs) improve cancer outcomes but may induce immune-related adverse events. Myasthenia gravis (MG), myositis, and myocarditis may co-occur as an overlap syndrome (“Triple-M”), but population-level data remain limited. This study aimed to characterize the pharmacovigilance profile, overlap patterns, and reported fatality of Triple-M associated with ICIs. **Methods**: A FAERS pharmacovigilance analysis was conducted using OpenVigil FDA and the openFDA API. Disproportionality metrics (ROR, PRR, χ^2^) were used to evaluate signals for MG, myositis, and myocarditis across nine ICIs. Triple-M was defined as the co-reporting of all three events and was evaluated over the study period from August 2016 to September 2025. **Results**: Among 272,753 ICI reports, 1395 (0.51%) MG, 3173 (1.16%) myocarditis, and 2018 (0.74%) myositis cases were identified; all nine ICIs met signal-detection criteria for all three toxicities (χ^2^ > 4). Pembrolizumab and nivolumab accounted for the highest absolute report counts, whereas nivolumab-relatlimab demonstrated the strongest disproportionality (ROR = 109.5 for MG, 106.4 for myocarditis, and 29.0 for myositis). Triple-M occurred in 114 unique reports (0.04% of all ICI-related adverse events), representing 8.2% of MG, 5.6% of myositis, and 3.6% of myocarditis cases. Co-reporting was common: among 5308 unique reports involving these toxicities, 1164 reports (21.9%) included at least two components of the triad. Triple-M cases were more common in men (56%), with a median age of 74 years (IQR 68–79), a median time-to-onset of 21 days (IQR 18–28), and 50% mortality among cases with available outcomes. **Conclusions**: Triple-M appears to be a severe overlap phenotype reported in association with immune checkpoint inhibitors, characterized by early onset, frequent co-reporting, and substantial reported fatality. Early recognition and coordinated multidisciplinary assessment may warrant further clinical evaluation and investigation of this overlap phenotype.

## 1. Introduction

Immune checkpoint inhibitors (ICIs) have revolutionized cancer care by improving survival across multiple malignancies, but can trigger immune-related adverse events (irAEs) affecting nearly every organ system [1,2,3,4]. Among these, myocarditis is one of the most feared because of its early onset and high mortality [5,6,7]. Despite increasing recognition, the reporting burden and overlap patterns of these toxicities remain poorly characterized. Severe neuromuscular irAEs, including myositis and myasthenia gravis (MG), have also emerged, often with rapid progression and respiratory compromise [8,9].

These toxicities arise from immune dysregulation induced by checkpoint inhibition. By inhibiting key regulatory pathways, including PD-1/PD-L1, CTLA-4, and LAG-3, ICIs enhance T-cell activation and antitumor immunity but may also disrupt self-tolerance, leading to off-target immune responses. The precise mechanisms underlying ICI-associated myocarditis, myositis, and MG remain incompletely understood and may differ by checkpoint target and treatment regimen. These events are generally considered immune-mediated and may involve loss of peripheral self-tolerance, autoreactive T-cell activation, and autoantibody-associated neuromuscular injury [4,10]. Because CTLA-4, PD-1/PD-L1, and LAG-3 blockade regulate immune activation through different pathways, the mechanisms and clinical expression of these toxicities may vary across ICI classes and between monotherapy and combination regimens [4,10].

Overlap between myositis and MG is increasingly recognized and is associated with poorer outcomes than isolated neuromuscular events [9]. Emerging evidence indicates that ICI-associated myocarditis occurs in approximately 1% of patients, representing a rare but clinically significant adverse event [5]. Similarly, myositis and MG are uncommon, with reported incidences of approximately 0.38% and 0.12%, respectively [8,11]. However, despite their low individual incidence, these conditions frequently co-occur; among patients with ICI-associated myocarditis, concurrent myositis and MG have been reported in approximately 25–40% and 10–11% of cases, respectively [12,13]. A particularly malignant phenotype involves concurrent myocarditis, myositis, and MG, recently termed “Triple-M syndrome,” with reported mortality of 40–60% [12,13,14,15]. However, clinical management of this syndrome remains heterogeneous, and current guidelines do not define Triple-M as a discrete entity [14,15,16,17,18]. Evidence is largely limited to case reports and small series, precluding reliable estimates of comparative reporting patterns, overlap burden, and reported lethality across ICI products [12,15,19].

Pharmacovigilance can address these gaps by enabling real-world signal detection at scale. Platforms such as OpenVigil FDA enable large-scale disproportionality analyses using the US FDA Adverse Event Reporting System (FAERS) [20,21]. Here, we report a pharmacovigilance analysis of Triple-M syndrome across all approved ICIs. The study objectives were to: (i) quantify disproportionality signals for MG, myositis, and myocarditis across ICIs; (ii) assess overlap and reported lethality within Triple-M syndrome; and (iii) describe the clinical features relevant to recognition and management.

## 2. Materials and Methods

### 2.1. Data Source and Case Identification

A retrospective pharmacovigilance analysis was performed using U.S. FAERS. Data were obtained through two complementary publicly accessible platforms: OpenVigil FDA [22], which provides deduplicated and standardized outputs optimized for disproportionality analyses, and the openFDA API [23], which enables custom, multi-term query construction and retrieval of raw case-level data.

FAERS was searched for all adverse event reports associated with all ICIs with U.S. FDA approval during the study period, including PD-1 inhibitors (nivolumab, pembrolizumab, and cemiplimab), LAG-3 inhibitor (relatlimab, typically used in combination with nivolumab), PD-L1 inhibitors (atezolizumab, durvalumab, and avelumab), and CTLA-4 inhibitors (ipilimumab and tremelimumab). Although ipilimumab received FDA approval in 2011, the first FAERS report describing Triple-M syndrome was submitted on 19 August 2016. Accordingly, for overlap analysis (Triple-M), the analytic window was defined as 19 August 2016, through the most recent FAERS update, September 2025. Disproportionality analyses for individual adverse events were based on all available FAERS reports within the database for each ICI–event pair.

All brand names were standardized to their corresponding generic names, and adverse events were identified using the Medical Dictionary for Regulatory Activities (MedDRA) Preferred Terms (PTs) ‘myasthenia gravis’, ‘myositis’, and ‘myocarditis’, as implemented in the FAERS database. Analyses were conducted at the PT level without post hoc aggregation; closely related PTs were retained as distinct entities to avoid subjective grouping and artificial signal inflation. The overlap phenotype (Triple-M syndrome) was defined as individual FAERS reports containing all three PTs simultaneously.

This study was designed and reported in accordance with the REporting of a Disproportionality Analysis for Drug Safety Signal Detection (READUS-PV) guidelines [24], and the selection and interpretation of disproportionality metrics followed established best-practice recommendations [25]. The completed READUS-PV checklist is provided in the Supplementary Materials (Supplementary Table S1).

### 2.2. Disproportionality Analysis

Disproportionality analyses were performed using OpenVigil FDA, which directly queries FAERS. Reports of MG, myositis, and myocarditis were retrieved for all nine approved immune checkpoint inhibitors (ICIs). For each drug–event pair, counts, frequencies, and signal metrics were extracted from OpenVigil FDA outputs, including the reporting odds ratio (ROR), proportional reporting ratio (PRR), chi-square (χ^2^), information component (IC), and Haldane odds ratio (HOR). A signal was considered present when the Evans criteria were met (≥3 cases, PRR > 2, and χ^2^ > 4). As the OpenVigil FDA does not support multiterm queries, the openFDA API was used to identify reports in which all three events (MG, myositis, and myocarditis) occurred concurrently. Reports were de-duplicated using unique case identifiers. Patient demographics, suspected agents, and outcomes were summarized using descriptive statistics. Time-to-onset (TTO) was calculated only when both therapy start (START_DT) and event onset (EVENT_DT or ONSET_DT) were available; implausible or negative intervals were excluded.

### 2.3. Statistical Analysis

For single adverse events, disproportionality measures (ROR, PRR, and chi-square) were derived from the OpenVigil outputs. For the Triple-M overlap, descriptive statistics were reported as counts and percentages. Mortality proportion was defined as the number of reports coded as “fatal” divided by the total number of reports, with sensitivity analyses restricted to reports with known outcomes. To evaluate the robustness of disproportionality signals to drug role coding, we repeated analyses using direct FAERS queries via the openFDA API, restricting to reports in which the immune checkpoint inhibitor was coded as a primary suspect (PS). The same case definitions and MedDRA preferred terms were applied. PS-restricted analyses were conducted using all available FAERS reports and compared with primary analyses, including all role codes.

Discrepancies between OpenVigil and API counts were due to differences in deduplication, MedDRA mapping, and drug name harmonization. All statistical analyses were performed using R, version 4.5.1, and IBM SPSS Statistics, version 30. This study used publicly available, de-identified data and did not require institutional review board approval.

## 3. Results

### 3.1. Overview of FAERS Reports

**Figure 1:**
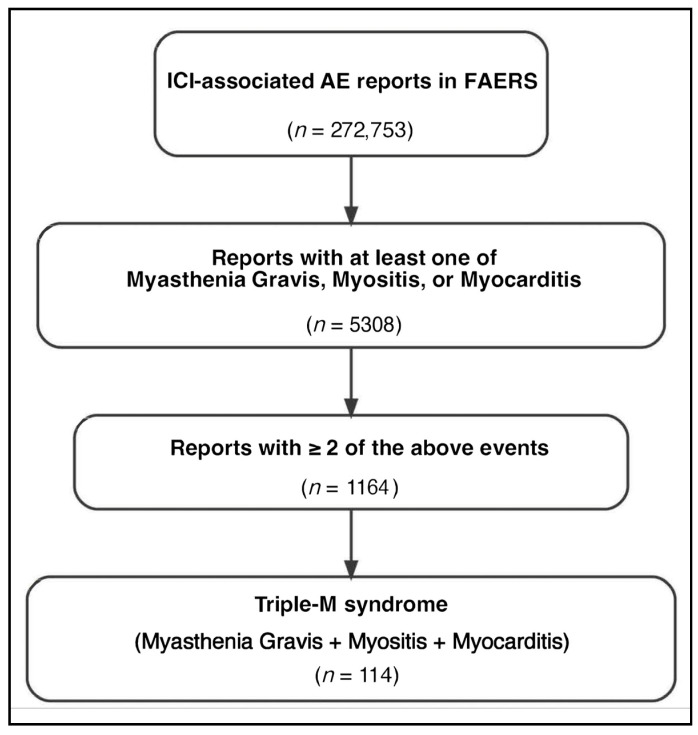
Workflow for identifying Triple-M (myasthenia gravis + myositis + myocarditis) cases from the FAERS database, 2016–2025.

Among the 272,753 ICI-associated adverse event (AE) reports retrieved from FAERS across all nine currently approved agents, 1395 (0.51%) MG, 2018 (0.74%) myositis, and 3173 (1.16%) myocarditis cases were identified. Table 1 summarizes their distribution by ICI, and Figure 1 outlines the process for case identification and inclusion.

### 3.2. Overlap and Co-Reported irAEs

**Figure 2:**
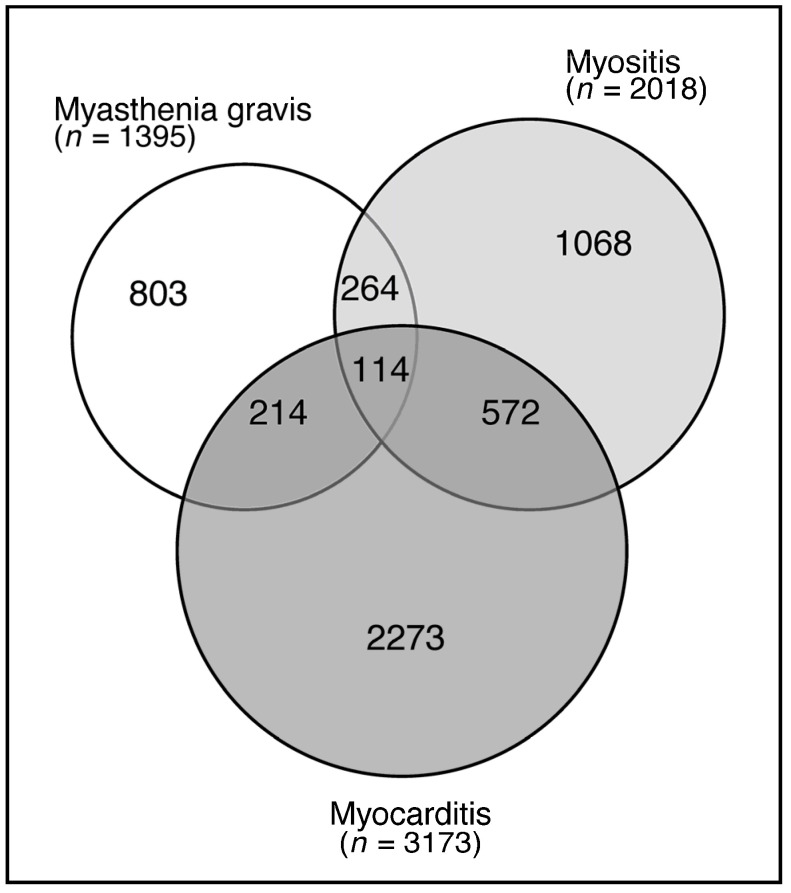
Overlap of immune checkpoint inhibitor (ICI)-associated myasthenia gravis, myositis, and myocarditis in FAERS reports.

Pairwise overlap was frequent: 27% of MG reports (*n* = 378) were co-reported with myositis, and 34% of myositis reports (*n* = 686) were co-reported with myocarditis. Overall, 1164 reports (22%) involved at least two of these toxicities, and 114 reports documented all three, constituting Triple-M syndrome (Figure 2).

Among the three index toxicities, the most frequently reported indications were melanoma, renal cell carcinoma, and non-small cell lung carcinoma. For each index event, the other two components of the triad were the most frequently co-reported toxicities, highlighting a consistent co-reporting pattern across the three toxicities. Detailed distributions of cancer indications and co-reported adverse events are provided in Table 2.

### 3.3. Myasthenia Gravis

**Figure 3:**
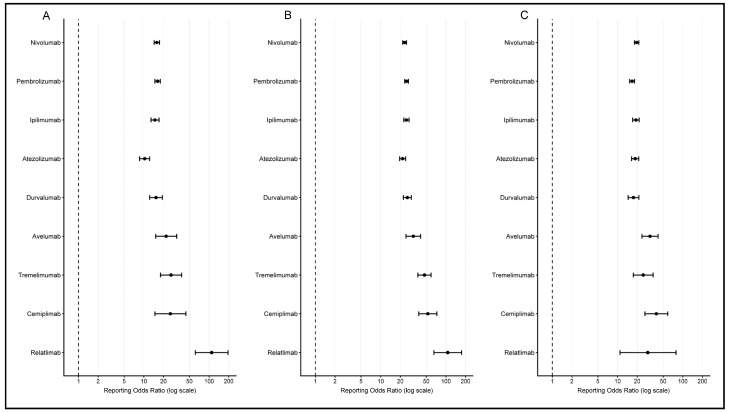
Forest plots showing reporting odds ratios (RORs) with 95% confidence intervals for (**A**) myasthenia gravis, (**B**) myocarditis, and (**C**) myositis associated with individual immune checkpoint inhibitors (ICIs). The vertical dashed line indicates ROR = 1.

A total of 1395 MG reports were identified across all ICIs. The highest reporting proportion occurred with nivolumab-relatlimab (3.65%). Although pembrolizumab (*n* = 460) and nivolumab (*n* = 457) accounted for the largest absolute report counts, their proportions were lower (0.53% and 0.51%, respectively). All agents met Evans’ signal-detection criteria (≥3 cases, PRR ≥ 2, χ^2^ ≥ 4), with nivolumab-relatlimab yielding the strongest disproportionality (ROR = 109.5 [95% CI 61.5–195.0]) (Figure 3A).

The median patient age was 73 years (IQR, 66–79 years), with male predominance (54.7%). The most common treatment indications were melanoma (*n* = 242), renal cell carcinoma (RCC; *n* = 238), and non-small cell lung carcinoma (NSCLC; *n* = 242). Details are provided in Table 3.

### 3.4. Myocarditis

Across all ICIs, 3173 myocarditis reports were identified. Pembrolizumab (*n* = 997) and nivolumab (*n* = 954) contributed more than 60% of the reports, reflecting widespread use. However, higher reporting proportions were observed for nivolumab–relatlimab (5.17%), cemiplimab (2.62%), and tremelimumab (2.33%). Nivolumab–relatlimab again showed the strongest disproportionality (ROR = 106 [95% CI 65.3–173.5]; Figure 3B). The median age was 71 years (IQR, 65–77 years). Detailed disproportionality metrics are summarized in Table 4.

### 3.5. Myositis

A total of 2018 myositis reports were identified. Nivolumab (671) and pembrolizumab (568) accounted for the largest absolute counts. In contrast, cemiplimab, with a reporting proportion of 1.61%, demonstrated the strongest disproportionality signal (ROR = 39.2 [95% CI 26.2–58.7]) (Figure 3C). The median age was 71 years (IQR, 65–79 years), with a male predominance (56.9%) (Table 5).

### 3.6. Triple-M Syndrome

Co-reporting of MG, myositis, and myocarditis occurred in 114 unique reports (0.04% of all ICI-related AEs), representing 8.2% of MG reports, 5.6% of myositis, and 3.6% of myocarditis (Figure 2). The median patient age was 74 years (IQR, 68–79 years), with a modest male predominance (57.9%, *n* = 66). The most frequently implicated regimens were pembrolizumab (*n* = 41), nivolumab (*n* = 21), nivolumab–ipilimumab (*n* = 24), and nivolumab–relatlimab (*n* = 5). Expanded regimen-level data are provided in Table 6.

**Figure 4:**
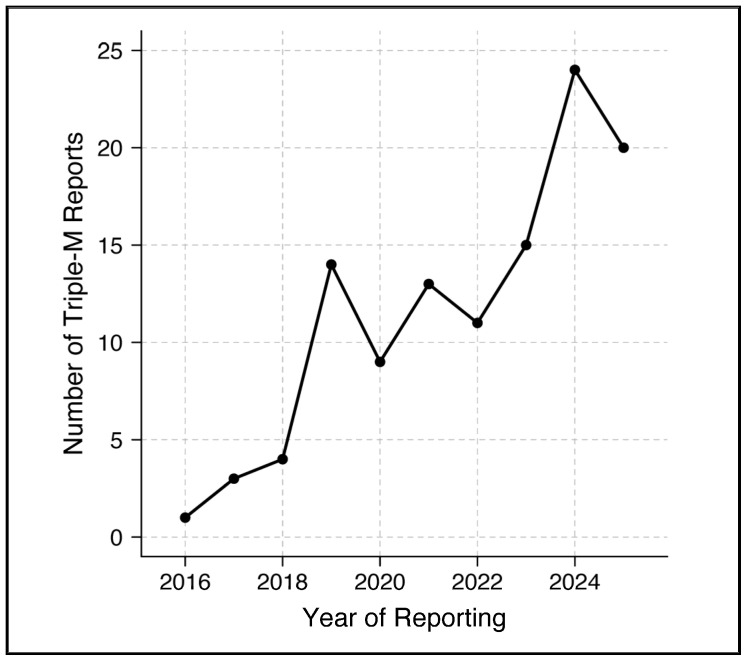
Temporal distribution of FAERS reports of immune checkpoint inhibitor (ICI)-associated Triple-M syndrome, 2016–2025. The number of reports increased from 1 in 2016 to 24 in 2024, consistent with rising use of ICI and increased awareness of overlap toxicity, rather than evidence of time-dependent or dose-dependent toxicity.

Overall, 35% of Triple-M reports were fatal, increasing to 50% among reports with known outcomes (*n* = 80). Most reports originated from the United States (39.5%) and Japan (31.6%), collectively accounting for over two-thirds of global submissions. The temporal pattern (Figure 4) demonstrated a steady rise in reported cases from 2016 (*n* = 1) to a peak in 2024 (*n* = 24), consistent with increased use of immune checkpoint inhibitors and greater recognition of overlapping immune-related toxicities. In the evaluable subset (*n* = 37, 32.5%), the median time to onset (TTO) was 21 days (IQR, 18–28), ranging from 4 to 59 days.

### 3.7. Sensitivity Analyses

PS-restricted analyses produced reporting proportions comparable to the primary analyses that included all role codes, with only minimal absolute differences across drugs and adverse events. These findings suggest that the observed signals were robust and not driven by inclusion of non-primary suspect reports. Detailed results are provided in Supplementary Tables S2–S4.

## 4. Discussion

Across more than 270,000 FAERS reports, MG, myositis, and myocarditis demonstrated disproportionality signals across all ICIs, supporting their recognition as clinically relevant irAEs that frequently co-occur rather than appearing exclusively in isolation [13,26]. Two complementary reporting patterns emerged. As expected, pembrolizumab and nivolumab, the most widely used ICIs, contributed the largest absolute numbers of Triple-M reports. In contrast, relatlimab (co-administered with nivolumab) and cemiplimab (primarily monotherapy) generated fewer reports but demonstrated much higher disproportionality, including RORs exceeding 100 for relatlimab-associated MG and myocarditis. Relatlimab’s strong signals may reflect differences in reporting patterns related to its recent introduction, including shorter time on market, lower cumulative exposure, and potential reporting bias; however, the magnitude of these signals suggests a robust reporting association in the context of dual immune checkpoint blockade, warranting further investigation.

Neuromuscular–cardiac overlap was frequent. Myositis co-occurred most often with both MG and myocarditis, as a dual syndrome or as a triad, suggesting it may be an early manifestation within the spectrum of muscle–cardiac involvement. Although less frequent in absolute count, myocarditis remains the most lethal complication, often accompanied by arrhythmias, conduction abnormalities, and heart failure [7,12,13,27]. MG, often perceived as isolated, appeared frequently as part of overlap syndromes, especially when respiratory failure or myocarditis were present. Overall, MG was reported in 1395 of 272,753 ICI-associated adverse-event reports (0.51%); however, among reports involving both myocarditis and myositis, MG was co-reported in 114 of 686 reports (16.6%), supporting enrichment of MG reporting within the myocarditis–myositis overlap phenotype.

Among 5308 unique reports of MG, myositis, or myocarditis, 21.9% co-reported ≥2 events, and 114 fulfilled Triple-M criteria. For each component adverse event, the other two components were the most frequently co-reported toxicities, suggesting a consistent pattern of co-reporting across these events. With myositis emerging as the central feature, this is consistent with previously reported antigenic overlap (e.g., anti-titin, anti-RYR, and anti-Kv1.4 antibodies) [10,19,27,28,29]. The most frequent co-reporting was observed between myositis and myocarditis (~25% of myositis cases). The absolute report count was highest for pembrolizumab and nivolumab; however, disproportionality was highest for cemiplimab, tremelimumab, and nivolumab–relatlimab. Clinically, identification of any single component may prompt early evaluation of the others (troponin, ECG, echocardiogram, creatine kinase, and assessment of respiratory and bulbar involvement), along with a multidisciplinary evaluation and consideration of immunosuppression. Guideline documents may benefit from recognizing Triple-M, particularly in the context of combination therapy.

The study’s findings align with previous case series, including a cohort of 60 patients with ICI-related myocarditis, myositis, and MG, that reported a 50% mortality and frequent early overlap [12]. Similarly, a second cohort of 50 patients described a median time-to-onset of 21 days—the equivalent duration of a single treatment cycle—and a 38% mortality rate [13]. Both reviews emphasized corticosteroids, intravenous immunoglobulin, and plasma exchange as the most frequently employed therapies; however, the outcomes remained poor. The present FAERS analysis extends these findings by quantifying disproportionality signals across approved ICIs, confirming that, while pembrolizumab and nivolumab account for the largest burden, relatlimab and cemiplimab show stronger disproportionality signals. Furthermore, the median time-to-onset of 21 days observed in previous studies was validated using the current pharmacovigilance dataset.

Among Triple-M cases with outcome data, reported fatality approached 50%. Combination regimens, including nivolumab–ipilimumab and nivolumab–relatlimab, accounted for a substantial proportion of deaths [6,29,30], suggesting additive or synergistic toxicity. Thus, neuromuscular and cardiac toxicities from ICIs should be recognized as clinically significant syndromes rather than rare events. Current ASCO, ESMO, and SITC guidelines list each AE individually but do not define Triple-M as a distinct overlap entity [16,17,18]. These data, together with prior cohorts [12,13,30], support greater clinical recognition of Triple-M and may help inform future guideline development, standardized monitoring strategies (e.g., serial creatine kinase, troponin, and autoantibody testing), and further investigation of management approaches, including immunosuppressive strategies. These findings may inform pharmacovigilance monitoring strategies, increase clinician awareness, and support further hypothesis generation. However, these observations should be interpreted in the context of potential reporting biases inherent to FAERS, including differences in ICI utilization, approved indications, market exposure, awareness of overlap toxicity, and geographic reporting patterns, which may influence the observed distribution and outcomes of Triple-M.

This study leveraged comprehensive FAERS data, integrating OpenVigil FDA and openFDA to maximize case capture and mitigate deduplication and nomenclature variability. Inclusion of all approved ICIs provided full therapeutic scope, and pairing disproportionality metrics with clinical outcome data provided both signal detection and real-world context. Our findings complement other recent FAERS-based oncology pharmacovigilance studies evaluating infectious complications across immune and targeted anti-cancer therapies; however, the present study focuses specifically on immune checkpoint inhibitor-associated immune-mediated overlap toxicities [31].

Limitations included reporting bias, under-reporting, incomplete clinical detail, and the absence of reliable denominators. Absolute reporting counts may be influenced by differences in time on market, utilization, and evolving clinical context across ICIs. However, disproportionality analyses reflect reporting patterns within the database rather than true incidence. As a result, causality cannot be inferred, and observed differences between agents should not be interpreted as true incidence or direct comparisons of risk. The completeness of FAERS reports varies across variables, and certain descriptive measures (e.g., time-to-onset and outcomes) were available only for a subset of cases. Restricting case identification to MedDRA Preferred Terms without term aggregation may have underestimated the number of relevant cases by excluding related or inconsistently coded terms. Discrepancies between OpenVigil and openFDA outputs may also occur due to differences in deduplication, MedDRA mapping, and drug name harmonization. Direct quantitative comparisons between platforms were not performed, which may introduce variability in absolute case counts. Mortality rates remain uncertain due to incomplete outcomes, and confounding by co-therapy, cancer type, patient characteristics, and reporting behavior cannot be excluded. Fatal outcomes may be overrepresented, as spontaneous reporting systems such as FAERS are more likely to capture severe or clinically significant events than milder cases.

Notably, sensitivity analyses restricted to primary suspect reports yielded consistent results across agents, supporting the robustness of the findings.

## 5. Conclusions

ICIs are consistently reported in association with myositis, myocarditis, and MG in FAERS, with relatlimab and cemiplimab showing stronger disproportionality signals despite less frequent use. These events frequently overlap, with Triple-M representing a highly lethal reported phenotype, particularly with combination therapy. Greater clinical recognition of Triple-M in oncology, neurology, and cardiology may support earlier multidisciplinary evaluation. Prospective studies and dedicated registries are needed to refine treatment and establish consensus management approaches.

## Figures and Tables

**Table 1:** Distribution of ICI-Associated Myasthenia Gravis, Myositis, and Myocarditis in FAERS.

Drug	Total Reports (*n*)	Myasthenia Gravis *n* (%)	Myocarditis *n* (%)	Myositis *n* (%)
Nivolumab	89,452	457 (0.51)	954 (1.07)	671 (0.75)
Pembrolizumab	87,584	460 (0.53)	997 (1.14)	568 (0.65)
Ipilimumab	40,122	199 (0.50)	481 (1.20)	307 (0.77)
Atezolizumab	33,789	118 (0.34)	359 (1.06)	254 (0.75)
Durvalumab	15,264	80 (0.52)	194 (1.27)	110 (0.72)
Avelumab	3720	28 (0.75)	59 (1.59)	48 (1.29)
Tremelimumab	3138	28 (0.89)	73 (2.33)	32 (1.02)
Cemiplimab	1488	13 (0.87)	39 (2.62)	24 (1.61)
Relatlimab *	329	12 (3.65)	17 (5.17)	4 (1.21)

* All relatlimab-associated reports in FAERS occurred in the context of nivolumab–relatlimab coadministration; no monotherapy cases were identified.

**Table 2:** Top ten cancer indications and most frequent co-reported adverse events associated with immune checkpoint inhibitor-related myasthenia gravis, myocarditis, and myositis (FAERS data through September 2025).

Index Event	Total Reports (*n*)	Top 10 Cancer Indications * (*n*)	Top 10 Co-Reported Adverse Events ** (*n*)
Myasthenia gravis	1395	Melanoma (242), RCC (238), NSCLC (242), TCC/UC (68), HCC (59), Endometrial cancer (54), GC (33), Mesothelioma (25), SCLC (22), BC (18)	Myositis (378), Myocarditis (328), Hepatitis (130), Immune-mediated myocarditis (94), Respiratory failure (91), Rhabdomyolysis (55), Pneumonitis (54), Encephalitis (43), GBS (41), Hypothyroidism (40)
Myocarditis	3173	NSCLC (664), Melanoma (567), RCC (444), HCC (174), BC (142), Endometrial cancer (90), Cervical cancer (36), TCC/UC (77), GC (59), CCA (28)	Myositis (686), Myasthenia gravis (328), Pneumonitis (309), Hepatitis (296), Colitis (272), Cardiac failure (155), Hypothyroidism (150), Atrioventricular complete block (121), Nephritis (115), Encephalitis (86)
Myositis	2018	Melanoma (464), NSCLC (410), RCC (255), HCC (129), HL (54), TCC/UC (64), Endometrial cancer (39), BC (34), SCLC (26), PC (24)	Myocarditis (686), Myasthenia gravis (378), Hepatitis (240), Pneumonitis (188), Colitis (182), Arthritis (117), Rash/Dermatitis (107), Encephalitis (95), Hypothyroidism (92), Rhabdomyolysis (83)

Abbreviations: BC, breast cancer; CCA, cholangiocarcinoma; GC, gastric cancer; GBS, Guillain–Barré syndrome; HCC, hepatocellular carcinoma; HL, Hodgkin lymphoma; NSCLC, non–small cell lung carcinoma; PC, prostate cancer; RCC, renal cell carcinoma; SCLC, small cell lung carcinoma; TCC/UC, transitional cell carcinoma/urothelial carcinoma. * Cancer indications were extracted directly from FAERS report fields and were summarized descriptively. ** Co-reported events reflect reporting associations and should not be interpreted as causal relationships. Only the ten most frequently reported indications and co-reported AEs are shown for conciseness.

**Table 3:** Pharmacovigilance Disproportionality Analysis of Myasthenia Gravis Reports Linked to Immune Checkpoint Inhibitor Therapy.

Drug	Reports (*n*)	Reporting Proportion (%DE/D)	χ^2^ (Yates)	PRR	ROR (95% CI)	HOR	IC	Sex	Median Age (IQR)
Nivolumab	457	0.51	5879.2	15.8	15.8 (14.4–17.4)	15.8	3.88	M 268 (58.6), F 118 (25.8), NA 71 (15.5)	70 (59–77)
Pembrolizumab	460	0.53	6107.3	16.2	16.3 (14.8–17.9)	16.3	3.92	M 268 (58.3), F 151 (32.8), NA 41 (8.9)	71 (65–77)
Ipilimumab	199	0.50	2460.7	14.7	14.8 (12.9–17.1)	14.8	3.84	M 116 (58.3), F 49 (24.6), NA 34 (17.1)	73 (64–79)
Atezolizumab	118	0.34	959.4	10.2	10.3 (8.6–12.3)	10.3	3.34	M 52 (44.1), F 34 (28.8), NA 32 (27.1)	72 (61–78)
Durvalumab	80	0.52	1043.9	15.4	15.39 (12.3–19.2)	15.49	3.92	M 25 (31.3), F 15 (18.8), NA 40 (50.0)	73 (68–77)
Avelumab	28	0.75	534.0	21.8	22.0 (15.2–31.9)	22.4	4.44	M 14 (50), F 7 (25), NA 7 (25)	75 (72–83)
Tremelimumab	28	0.89	642.8	25.9	26.1 (18.0–37.9)	26.6	4.69	M 7 (25), F 5 (17.9), NA 16 (57.1)	73 (64–79)
Cemiplimab	13	0.87	279.1	25.3	25.5 (14.8–44.1)	26.5	4.66	M 6 (46.2), F 2 (15.4), NA 5 (38.4)	72 (69–80)
Relatlimab *	12	3.65	1139.1	105.6	109.5 (61.5–195.0)	113.9	6.72	M 6 (50.0), F 5 (41.7), NA 1 (8.3)	76 (67–85)
Total	1395	--	--	--	--	--	--	M 762 (54.7), F 386 (27.7), NA 247 (17.7)	73 (66–79)

Abbreviations: CI, confidence interval; DE/D, drug event/drug; HOR, high-order reporting odds ratio; IC, information component; IQR, interquartile range; PRR, proportional reporting ratio; ROR, reporting odds ratio. All listed ICIs fulfilled Evans’ pharmacovigilance signal detection criteria (≥3 cases, PRR ≥ 2, χ^2^ ≥ 4). ICI classes/functions: nivolumab, pembrolizumab, and cemiplimab are PD-1 inhibitors; atezolizumab, durvalumab, and avelumab are PD-L1 inhibitors; ipilimumab and tremelimumab are CTLA-4 inhibitors; relatlimab is a LAG-3 inhibitor. * All relatlimab-associated reports in FAERS occurred in the context of nivolumab–relatlimab coadministration; no monotherapy cases were identified.

**Table 4:** Pharmacovigilance Disproportionality Analysis of Myocarditis Reports Linked to Immune Checkpoint Inhibitor Therapy.

Drug	Reports (*n*)	Reporting Proportion (%DE/D)	χ^2^ (Yates)	PRR	ROR (95% CI)	HOR	IC	Sex	Median Age (IQR)
Nivolumab	954	1.07	18,063.4	22.9	23.2 (21.7–24.8)	23.2	4.38	M 549 (57.6), F 258 (27.0), NA 147 (15.4)	69 (63–76)
Pembrolizumab	997	1.14	20,276.9	24.6	24.9 (23.3–26.6)	24.9	4.47	M 405 (40.6), F 490 (49.2), NA 102 (10.2)	71 (65–77)
Ipilimumab	481	1.20	10,314.6	24.53	24.8 (22.6–27.2)	24.84	4.55	M 267 (55.5), F 134 (27.9), NA 80 (16.6)	71 (66–77)
Atezolizumab	359	1.06	6737.4	21.5	21.7 (19.5–24.1)	21.72	4.37	M 155 (43.2), F 92 (25.6), NA 112 (31.2)	71 (66–76)
Durvalumab	194	1.27	4412.9	25.3	25.6 (22.2–29.5)	25.65	4.63	M 76 (39.2), F 28 (14.4), NA 90 (46.4)	67 (60–74)
Avelumab	59	1.59	1681.2	31.1	31.6 (24.4–40.9)	31.87	4.95	M 28 (47.5), F 7 (11.9), NA 24 (40.6)	69 (67–72)
Tremelimumab	73	2.33	3127.1	45.7	46.8 (37.1–59.1)	47.1	5.50	M 17 (23.3), F 18 (24.7), NA 38 (52.0)	67 (60–74)
Cemiplimab	39	2.62	1868.6	51.3	52.7 (38.3–72.5)	53.35	5.68	M 19 (48.7), F 5 (12.8), NA 15 (38.5)	75 (72–83)
Relatlimab *	17	5.17	1582.7	101.0	106.4 (65.3–173.5)	109.39	6.66	M 5 (29.4), F 7 (41.2), NA 5 (29.4)	76 (69–81)
Total	3173	--	--	--	--	--	--	M 1521 (47.9), F 1039 (32.8), NA 613 (19.3)	71 (65–77)

Abbreviations: CI, confidence interval; DE/D, drug event/drug; HOR, high-order reporting odds ratio; IC, information component; IQR, interquartile range; PRR, proportional reporting ratio; ROR, reporting odds ratio. All listed ICIs fulfilled Evans’ pharmacovigilance signal detection criteria (≥3 cases, PRR ≥ 2, χ^2^ ≥ 4). ICI classes/functions: nivolumab, pembrolizumab, and cemiplimab are PD-1 inhibitors; atezolizumab, durvalumab, and avelumab are PD-L1 inhibitors; ipilimumab and tremelimumab are CTLA-4 inhibitors; relatlimab is a LAG-3 inhibitor. * All relatlimab-associated reports in FAERS occurred in the context of nivolumab–relatlimab coadministration; no monotherapy cases were identified.

**Table 5:** Pharmacovigilance Disproportionality Analysis of Myositis Reports Linked to Immune Checkpoint Inhibitor Therapy.

Drug	Reports (*n*)	Reporting Proportion (%DE/D)	χ^2^ (Yates)	PRR	ROR (95% CI)	HOR	IC	Sex	Median Age (IQR)
Nivolumab	671	0.75	10,741.3	19.4	19.6 (18.1–21.2)	19.6	4.16	M 443 (66.0), F 152 (22.6), NA 76 (11.3)	71 (64–80)
Pembrolizumab	568	0.65	7712.8	16.6	16.7 (15.3–18.2)	16.7	3.95	M 304 (53.5), F 198 (34.9), NA 66 (11.6)	71 (65–77)
Ipilimumab	307	0.77	5002.5	18.9	19.1 (17.0–21.4)	19.1	4.19	M 186 (60.6), F 75 (24.4), NA 46 (15.0)	69 (58–79)
Atezolizumab	254	0.75	4053.4	18.5	18.6 (16.4–21.1)	18.6	4.16	M 122 (48.0), F 54 (21.3), NA 78 (30.7)	75 (68–83)
Durvalumab	110	0.72	1663.5	17.4	17.5 (14.5–21.2)	17.5	4.10	M 53 (48.2), F 10 (9.1), NA 47 (42.7)	71 (63–79)
Avelumab	48	1.29	1354.4	31.0	31.4 (23.6–41.7)	31.4	4.94	M 23 (47.9), F 11 (22.9), NA 14 (29.2)	76 (67–86)
Tremelimumab	32	1.02	693.1	24.4	24.7 (17.4–35.0)	25.0	4.60	M 8 (25.0), F 7 (21.9), NA 17 (53.1)	65 (44–78)
Cemiplimab	24	1.61	839.5	38.6	39.2 (26.2–58.7)	40.0	5.27	M 10 (41.7), F 2 (8.3), NA 12 (50)	77 (72–83)
Relatlimab *	4	1.21	82.1	29.0	29.0 (10.9–78.7)	32.9	4.8	F 3 (75.0), NA 1 (25.0)	83.5 (76–91)
Total	2018	--	--	--	--	--	--	M 1149 (56.9), F 512 (25.4), NA 357 (17.7)	71 (65–79)

Abbreviations: CI, confidence interval; DE/D, drug event/drug; HOR, high-order reporting odds ratio; IC, information component; IQR, interquartile range; PRR, proportional reporting ratio; ROR, reporting odds ratio. All listed ICIs fulfilled Evans’ pharmacovigilance signal detection criteria (≥3 cases, PRR ≥ 2, χ^2^ ≥ 4). ICI classes/functions: nivolumab, pembrolizumab, and cemiplimab are PD-1 inhibitors; atezolizumab, durvalumab, and avelumab are PD-L1 inhibitors; ipilimumab and tremelimumab are CTLA-4 inhibitors; relatlimab is a LAG-3 inhibitor. * All relatlimab-associated reports in FAERS occurred in the context of nivolumab–relatlimab coadministration; no monotherapy cases were identified.

**Table 6:** Triple-M Syndrome Cases by Regimen (*n* = 114).

Drug/s	Cases *n* (%)	Median Age (IQR)	Sex *n* (%)	Fatal *n* (%)	Recovered *n* (%)	Sequelae *n* (%)	Unknown *n* (%)
Pembrolizumab	41 (36.0)	77 (68–81)	M 27 (65.9), F 13 (31.7), NA 1 (2.4)	14 (34.1)	11 (26.8)	4 (9.8)	12 (29.3)
Nivolumab	21 (18.4)	73 (64–78)	M 17 (81.0), F 4 (19.0), NA 0 (0%)	9 (42.9)	5 (23.8)	3 (14.3)	4 (19.0)
Cemiplimab	3 (2.6)	71 (70–71)	M 2 (66.7), F 0 (0.0), NA 1 (33.3)	2 (66.7)	0 (0.0)	0 (0.0)	1 (33.3)
Atezolizumab	7 (6.1)	83 (77–83)	M 4 (57.1), F 3 (42.9), NA 0 (0.0)	0 (0.0)	3 (42.9)	0 (0.0)	4 (57.1)
Durvalumab	3 (2.6)	77 (76–78)	M 0 (0.0), F 1 (33.3), NA 2 (66.7)	2 (66.7)	0 (0.0)	0 (0.0)	1 (33.3)
Avelumab	3 (2.6)	42 (25–59)	M 2 (66.7), F 1 (33.3), NA 0 (0.0)	1 (33.3)	2 (66.7)	0 (0.0)	0 (0.0)
Nivolumab + Ipilimumab	24 (21.1)	73 (68–76)	M 14 (58.3), F 9 (37.5), NA 1 (4.2)	5 (20.8)	8 (33.3)	2 (8.3)	9 (37.5)
Nivolumab + Relatlimab	5 (4.4)	76 (76–83)	M 0 (0.0), F 4 (80.0), NA 1 (20.0)	2 (40.0)	1 (20.0)	1 (20.0)	1 (20.0)
Avelumab + Nivolumab	1 (0.9)	NA	M 0 (0.0), F 0 (0.0), NA 1 (100)	0 (0.0)	0 (0.0)	0 (0.0)	1 (100.0)
Multiple ICIs (≥3 agents)	6 (5.3)	NA	M 0 (0.0), F 0 (0.0), NA 6 (100)	5 (83.3)	0 (0.0)	0 (0.0)	1 (16.7)
TOTAL	114 (100)	74 (68–79)	M 66 (57.9), F 35 (30.7), NA 13 (11.4)	40 (35.1)	30 (26.3)	10 (8.8)	34 (29.8)

Sequelae = Patient survived with residual side effects.

## Data Availability

Data used in this study are publicly available from the U.S. Food and Drug Administration Adverse Event Reporting System (FAERS). Data were accessed via OpenVigil FDA and the openFDA API on 1 October 2025. The aggregate data supporting the main analyses are provided in the manuscript and Supplementary Tables S2–S4.

## Supplementary Materials

The following supporting information can be downloaded at https://www.mdpi.com/article/10.3390/healthcare14111466/s1, Table S1: Completed READUS-PV reporting checklist for the disproportionality analysis; Table S2: Sensitivity analysis restricted to the primary suspect (PS) role for myasthenia gravis; Table S3: Sensitivity analysis restricted to the primary suspect (PS) role for myocarditis; Table S4: Sensitivity analysis restricted to the primary suspect (PS) role for myositis.

## Abbreviations

The following abbreviations are used in this manuscript:

AE	Adverse event
BC	Breast cancer
CCA	Cholangiocarcinoma
CI	Confidence interval
DE/D	Drug event/drug
FAERS	Food and Drug Administration Adverse Event Reporting System
GC	Gastric cancer
GBS	Guillain–Barré syndrome
HCC	Hepatocellular carcinoma
HL	Hodgkin lymphoma
HOR	High-order reporting odds ratio
IC	Information component
ICI	Immune checkpoint inhibitor
irAE	Immune-related adverse event
IQR	Interquartile range
MG	Myasthenia gravis
MedDRA	Medical Dictionary for Regulatory Activities
NSCLC	Non–small cell lung carcinoma
PC	Prostate cancer
PRR	Proportional reporting ratio
RCC	Renal cell carcinoma
ROR	Reporting odds ratio
SCLC	Small cell lung carcinoma
TCC/UC	Transitional cell carcinoma/urothelial carcinoma
TTO	Time to onset
US	United States
WHO	World Health Organization
χ^2^	Chi-squared test
Triple-M	Myasthenia gravis + Myositis + Myocarditis overlap syndrome

## References

[1] Sharma P., Allison J.P. (2015). Immune checkpoint targeting in cancer therapy: Toward combination strategies with curative potential. Cell.

[2] Topalian S.L., Drake C.G., Pardoll D.M. (2015). Immune checkpoint blockade: A common denominator approach to cancer therapy. Cancer Cell.

[3] Robert C. (2020). A decade of immune-checkpoint inhibitors in cancer therapy. Nat. Commun..

[4] Postow M.A., Sidlow R., Hellmann M.D. (2018). Immune-related adverse events associated with immune checkpoint blockade. N. Engl. J. Med..

[5] Mahmood S.S., Fradley M.G., Cohen J.V., Nohria A., Reynolds K.L., Heinzerling L.M., Sullivan R.J., Damrongwatanasuk R., Chen C.L., Gupta D. (2018). Myocarditis in patients treated with immune checkpoint inhibitors. J. Am. Coll. Cardiol..

[6] Salem J.E., Manouchehri A., Moey M., Lebrun-Vignes B., Bastarache L., Pariente A., Gobert A., Spano J.-P., Balko J.M., Bonaca M.P. (2018). Cardiovascular toxicities associated with immune checkpoint inhibitors: An observational, retrospective, pharmacovigilance study. Lancet Oncol..

[7] Jensen G., Wang X., Kuempel J., Palaskas N., Chen Z., Yu W., Chen Y., Mohammad H., Luo W., Chang J. (2025). Immune checkpoint inhibitor-associated myocarditis: A historical and comprehensive review. Am. J. Physiol. Heart Circ. Physiol..

[8] Suzuki S., Ishikawa N., Konoeda F., Seki N., Fukushima S., Takahashi K., Uhara H., Hasegawa Y., Inomata S., Otani Y. (2017). Nivolumab-related myasthenia gravis with myositis and myocarditis in Japan. Neurology.

[9] Moreira A., Loquai C., Pföhler C., Kähler K.C., Knauss S., Heppt M.V., Gutzmer R., Dimitriou F., Meier F., Mitzel-Rink H. (2019). Myositis and neuromuscular side-effects induced by immune checkpoint inhibitors. Eur. J. Cancer.

[10] Albarrán V., Chamorro J., Rosero D.I., Saavedra C., Soria A., Carrato A., Gajate P. (2022). Neurologic toxicity of immune checkpoint inhibitors: A review of literature. Front. Pharmacol..

[11] Hamada N., Maeda A., Takase-Minegishi K., Kirino Y., Sugiyama Y., Namkoong H., Horita N., Yoshimi R., Nakajima H., YCU irAE Working Group (2021). Incidence and Distinct Features of Immune Checkpoint Inhibitor-Related Myositis from Idiopathic Inflammatory Myositis: A Single-Center Experience With Systematic Literature Review and Meta-Analysis. Front. Immunol..

[12] Pathak R., Katel A., Massarelli E., Villaflor V.M., Sun V., Salgia R. (2021). Immune checkpoint inhibitor–induced myocarditis with myositis/myasthenia gravis overlap syndrome: A systematic review of cases. Oncologist.

[13] Lipe D.N., Qdaisat A., Krishnamani P.P., Nguyen T.D., Chaftari P., El Messiri N., Srinivasan A., Galvis-Carvajal E., Reyes-Gibby C.C., Wattana M.K. (2024). Myocarditis, Myositis, and Myasthenia Gravis Overlap Syndrome Associated with Immune Checkpoint Inhibitors: A Systematic Review. Diagnostics.

[14] Furlepa M., Watts I., Carr A.S. (2025). Management of Triple M syndrome: A narrative review of immune checkpoint inhibitor-induced myasthenia gravis, myositis and myocarditis. Cancers.

[15] Sánchez-Camacho A., Torres-Zurita A., Gallego-López L., Hernández-Pacheco R., Silva-Romeiro S., Álamo de la Gala M.C., de Ceballos E.P.-G., de la Cruz-Merino L. (2025). Management of immune-related myocarditis, myositis and myasthenia gravis (MMM) overlap syndrome: A single-institution case series and literature review. Front. Immunol..

[16] Puzanov I., Diab A., Abdallah K., Bingham C.O., Brogdon C., Dadu R., Hamad L., Kim S., Lacouture M.E., LeBoeuf N.R. (2017). Managing toxicities associated with immune checkpoint inhibitors: Consensus recommendations from the Society for Immunotherapy of Cancer (SITC) Toxicity Management Working Group. J. Immunother. Cancer.

[17] Haanen J.B.A.G., Carbonnel F., Robert C., Kerr K.M., Peters S., Larkin J., Jordan K. (2017). Management of toxicities from immunotherapy: ESMO clinical practice guidelines for diagnosis, treatment and follow-up. Ann. Oncol..

[18] Brahmer J.R., Lacchetti C., Schneider B.J., Atkins M.B., Brassil K.J., Caterino J.M., Chau I., Ernstoff M.S., Gardner J.M., Ginex P. (2018). Management of immune-related adverse events in patients treated with immune checkpoint inhibitor therapy: American Society of Clinical Oncology clinical practice guideline. J. Clin. Oncol..

[19] Arora P., Talamo L., Dillon P., Gentzler R.D., Millard T., Salerno M., Slingluff C.L., Gaughan E.M. (2020). Severe combined cardiac and neuromuscular toxicity from immune checkpoint blockade: An institutional case series. Cardio-Oncology.

[20] Sakaeda T., Tamon A., Kadoyama K., Okuno Y. (2013). Data mining of the public version of the FDA adverse event reporting system. Int. J. Med. Sci..

[21] Böhm R., Von Hehn L., Herdegen T., Klein H.J., Bruhn O., Petri H., Höcker J. (2016). OpenVigil FDA. Inspection of US American adverse drug events pharmacovigilance data and novel clinical applications. PLoS ONE.

[22] OpenVigil Open Tools for Data Mining and Analysis of Pharmacovigilance Data. https://openvigil.sourceforge.net/.

[23] U.S. Food and Drug Administration openFDA Application Programming Interface (API). https://open.fda.gov/apis/.

[24] Fusaroli M., Salvo F., Bégaud B., AlShammari T.M., Bate A., Battini V., Brueckner A., Candore G., Carnovale C., Crisafulli S. (2024). The REporting of A Disproportionality Analysis for DrUg Safety Signal Detection Using Individual Case Safety Reports in PharmacoVigilance (READUS-PV): Explanation and elaboration. Drug Saf..

[25] Cutroneo P.M., Sartori D., Tuccori M., Crisafulli S., Battini V., Carnovale C., Rafaniello C., Capuano A., Poluzzi E., Moretti U. (2024). Conducting and interpreting disproportionality analyses derived from spontaneous reporting systems. Front. Drug Saf. Regul..

[26] Yin Q., Wu L., Han L., Zheng X., Tong R., Li L., Bai L., Bian Y. (2023). Immune-related adverse events of immune checkpoint inhibitors: A review. Front. Immunol..

[27] Vicino A., Hottinger A.F., Latifyan S., Boughdad S., Becce F., Prior J.O., Kuntzer T., Brouland J.-P., Dunet V., Obeid M. (2024). Immune checkpoint inhibitor-related myositis and myocarditis: Diagnostic pitfalls and imaging contribution in a real-world, institutional case series. J. Neurol..

[28] Suzuki S., Nagane Y., Uzawa A., Imai T., Murai H., Nakahara J., Utsugisawa K. (2020). Anti-striational antibodies: Expanding their clinical significance. Clin. Exp. Neuroimmunol..

[29] Moslehi J.J., Salem J.E., Sosman J.A., Lebrun-Vignes B., Johnson D.B. (2018). Increased reporting of fatal immune checkpoint inhibitor-associated myocarditis. Lancet.

[30] Johnson D.B., Nebhan C.A., Moslehi J.J., Balko J.M. (2022). Immune-checkpoint inhibitors: Long–term implications of toxicity. Nat. Rev. Clin. Oncol..

[31] Alsowaida Y.S., Alsolami A., Almangour T.A., Abraham I. (2025). Infectious complications associated with immune and targeted anti-cancer therapies: A retrospective study of the FDA adverse events reporting system (FAERS). Expert Opin. Drug Saf..

