# Supplementary material for: Triple-M Overlap Syndrome Associated with Immune Checkpoint Inhibitors: A FAERS Pharmacovigilance Analysis
Source: Healthcare (Basel). 2026 May 26;14(11):1466. doi: 10.3390/healthcare14111466 (PMC13257212; doi:10.3390/healthcare14111466)
Supplement: Supplementary file 1 [file healthcare-14-01466-s001.zip › healthcare-4241635-supplementary.pdf]

Supplementary Material

Triple-M Overlap Syndrome Associated with Immune Checkpoint Inhibitors: A FAERS Pharmacovigilance Analysis

Bader Alshamsan[1], Terry L. Ng[2,3,4]

Supplementary Table S1. Completed READUS-PV reporting checklist for the disproportionality analysis

Section and topic	Item #	Checklist item	Location where item is reported
**Title**			
	1a	*If disproportionality analyses are a prominent component of the published study, the study should be identified as a "disproportionality analysis". The type of data and name of the database(s) should be specified.*	Title; Abstract – Methods (Lines 1–5)
	1b	*Report the name of adverse event(s) and/or drug(s) under study, when applicable.*	Methods 2.1; Tables 1–6
**Introduction**			
Background	*2a*	*Describe the drug(s) and its utilization, the nature of the adverse event(s) under study and its frequency, and the existing knowledge on the drug-event combination.*	Introduction paragraphs 1–3
	2b	*Specify the rationale for performing the analysis, e.g., as part of routine pharmacovigilance, to investigate an overall safety profile, or to assess a pre-specified hypothesis.*	Introduction paragraph 3
	2c	*Explain why ICSR databases and disproportionality analysis are suitable to fill the knowledge gap.*	Introduction paragraphs 3–4 ; and Methods 2.1
Objectives	*3*	*State specific objectives, identifying the adverse event(s), the drug(s), and the reference group, including any pre-specified hypothesis, if applicable.*	Introduction final paragraph
**Methods**			
Study design	*4a*	*Identify the study (i.e., "disproportionality analysis") and the type of data used (e.g., "individual case safety reports").*	Methods 2.2
	4b	*Provide an outline of the entire study design, including primary and sensitivity analyses performed, and other designs such as case-by-case analysis or literature review.*	Methods 2.1, and 2.2 ; Figure 1

Data description, access, and pre-processing	*5a*	*Specify the name of the database(s), the database(s) custodian, and the coverage. Specify the type/number of drugs included within the database and the thesaurus, taxonomies, or ontologies used for coding drugs and events.*	Methods 2.1,
	5b	*Specify the extraction dates and describe and justify all choices used for data pre-processing, including any data transformation or exclusion, if appropriate.*	Methods 2.1, and 2.3
Variables definition	*6a*	*Describe the study population, including any restriction.*	Methods 2.1, and Figure 1
	6b	*Describe the nature and the meaning of key variables assessed in the work.*	Methods 2.3 & Results 3.6; Table 6
	6c	*Specify and justify any grouping of drugs or events. For drugs, specify and justify whether active ingredients/trade names/salts were considered and/or the selected role.*	Methods 2.1; Tables 1 & 6 footnotes
	6d	*Describe any additional data source used, the type of data, and how they interact with ICSRs.*	Methods 2.1, and 2.2
Statistical methods	*7a*	*Present any descriptive analysis performed, specifying variables investigated, statistical tests, and significance thresholds.*	Methods 2.3, and Tables 3–5
	7b	*Describe the measure(s) selected for the disproportionality analysis including any threshold used to identify signals of disproportionate reporting. Explain the reason for this choice if applicable.*	Methods 2.3, and Tables 3–5
	7c	*Clearly describe any sensitivity analysis and any tool to control confounding, including any restriction, subgroup, stratification, adjustment, or interaction.*	Methods 2.1, and 2.3
	7d	*Specify the variables and methods used for the case-by-case analysis, including any algorithm or criteria used to assess causality, if performed.*	Methods 2.3
	7e	*Specify any statistical methods used for other data sources.*	Methods 2.3
**Results**			
Participants	*8a*	*Specify the number of individual case safety reports included at each stage, including reasons for exclusion.*	Results 3.1–3.2; Figure 1
	8b	*Provide key demographic and clinical characteristics of cases, if possible comparing cases with any appropriate reference group.*	Results 3.6; Table 6; MG/Myositis/Myocarditis tables
Disproportionality analysis	*9*	*Present all results including confidence intervals. Present also results of sensitivity analyses, if performed.*	Results 3.3–3.5; Figure 3 forest plots
Case-by-case analysis	*10*	*Present the case-by-case analysis of key variables. Present the causality assessment, if applicable.*	Not applicable
**Discussion**			
Key results	*11*	*Discuss key results with reference to study objectives and contextualize them within the current literature and other consulted sources. Clearly discriminate between expected reactions and emerging safety signals.*	Discussion paragraphs 1–4
External validity	*12a*	*Discuss the external validity of the results to the general population.*	Discussion paragraph 5

	12b	*Discuss the potential relevance of results in clinical practice*	Discussion paragraphs 4–6
	12c	*Propose further study designs if applicable*	Conclusion paragraph 2
Limitations	*13*	*Present general limitations, making clear that disproportionality analysis alone cannot prove causation or measure incidence, and specific limitations, including confounding and reporting bias and efforts to mitigate them.*	Discussion – Limitations paragraph
**Declarations**			
	14a	*Provide the source of funding/sponsorship and the role of the funders/sponsors for the present study and for any original study on which the present article is based.*	Funding Statement
	14b	*Clearly identify potential commercial and intellectual conflicts of interest (e.g., link to any drug/event investigated, whether financial, legal action, or software used).*	COI Statement
	14c	*Declare any institutional approval needed or granted in the investigation.*	IRB / Ethics Statement
	14d	*Include a statement on data availability, code availability (including the version of the statistical software used), and protocol registration.*	Data Availability + Methods software version

Supplementary Table S2. Sensitivity analysis restricted to the primary suspect (PS) role for myasthenia gravis

Drug	All-role reports *n*/N (%)	PS-restricted reports *n*/N (%)
Atezolizumab	70/19,611 (0.36)	70/19,607 (0.36)
Avelumab	18/2141 (0.84)	18/2141 (0.84)
Cemiplimab	15/1118 (1.34)	15/1115 (1.35)
Durvalumab	56/6447 (0.87)	56/6446 (0.87)
Ipilimumab	125/16,174 (0.77)	125/16,169 (0.77)
Nivolumab	289/43,137 (0.67)	289/43,106 (0.67)
Pembrolizumab	207/28,619 (0.72)	205/28,558 (0.72)
Relatlimab	10/401 (2.49)	10/401 (2.49)

Tremelimumab	27/2,172 (1.24)	27/2,172 (1.24)

Supplementary Table S3. Sensitivity analysis restricted to the primary suspect (PS) role for myocarditis

Drug	All-role reports *n*/N (%)	PS-restricted reports *n*/N (%)
Atezolizumab	291/19,611 (1.48)	291/19,607 (1.48)
Avelumab	47/2141 (2.20)	47/2141 (2.20)
Cemiplimab	34/1118 (3.04)	34/1115 (3.05)
Durvalumab	162/6447 (2.51)	162/6446 (2.51)
Ipilimumab	446/16174 (2.76)	446/16,169 (2.76)
Nivolumab	868/43,137 (2.01)	867/43,106 (2.01)
Pembrolizumab	665/28,619 (2.32)	665/28,558 (2.33)
Relatlimab	28/401 (6.98)	28/401 (6.98)
Tremelimumab	73/2172 (3.36)	73/2172 (3.36)

Supplementary Table S4. Sensitivity analysis restricted to the primary suspect (PS) role for myositis

Drug	All-role reports *n*/N (%)	PS-restricted reports *n*/N (%)
Atezolizumab	183/19,611 (0.93)	183/19,607 (0.93)
Avelumab	39/2141 (1.82)	39/2141 (1.82)
Cemiplimab	25/1118 (2.24)	25/1115 (2.24)
Durvalumab	97/6447 (1.50)	97/6446 (1.50)
Ipilimumab	251/16,174 (1.55)	251/16,169 (1.55)

Nivolumab	561/43,137 (1.30)	561/43,106 (1.30)
Pembrolizumab	342/28,619 (1.20)	341/28,558 (1.19)
Relatlimab	14/401 (3.49)	14/401 (3.49)
Tremelimumab	32/2172 (1.47)	32/2172 (1.47)
